# ACSL4 promotes prostate cancer growth, invasion and hormonal resistance

**DOI:** 10.18632/oncotarget.6438

**Published:** 2015-11-30

**Authors:** Xinyu Wu, Fangming Deng, Yirong Li, Garrett Daniels, Xinxin Du, Qinghu Ren, Jinhua Wang, Ling Hang Wang, Yang Yang, Valerio Zhang, David Zhang, Fei Ye, Jonathan Melamed, Marie E. Monaco, Peng Lee

**Affiliations:** ^1^ Department of Pathology, New York University School of Medicine, New York, NY, USA; ^2^ Department of Pediatrics, New York University School of Medicine, New York, NY, USA; ^3^ NYU Cancer Institute, New York University School of Medicine, New York, NY, USA; ^4^ NYU Center for Health Informatics and Bioinformatics, New York University School of Medicine, New York, NY, USA; ^5^ Department of Pathology, Mount Sinai School of Medicine, New York, NY, USA; ^6^ Department of Neuroscience and Physiology, New York University School of Medicine, New York, NY, USA; ^7^ Department of Urology, New York University School of Medicine, New York, NY, USA; ^8^ VA New York Harbor Healthcare System, New York University School of Medicine, New York, NY, USA

**Keywords:** androgen receptor, prostate cancer, ACSL4, castration resistance

## Abstract

Increases in fatty acid metabolism have been demonstrated to promote the growth and survival of a variety of cancers, including prostate cancer (PCa). Here, we examine the expression and function of the fatty acid activating enzyme, long-chain fatty acyl-CoA synthetase 4 (ACSL4), in PCa. Ectopic expression of ACSL4 in ACSL4-negative PCa cells increases proliferation, migration and invasion, while ablation of ACSL4 in PCa cells expressing endogenous ACSL4 reduces cell proliferation, migration and invasion. The cell proliferative effects were observed both *in vitro*, as well as *in vivo.* Immunohistochemical analysis of human PCa tissue samples indicated ACSL4 expression is increased in malignant cells compared with adjacent benign epithelial cells, and particularly increased in castration-resistant PCa (CRPC) when compared with hormone naive PCa. In cell lines co-expressing both ACSL4 and AR, proliferation was independent of exogenous androgens, suggesting that ACSL4 expression may lead to CRPC. In support for this hypothesis, ectopic ACSL4 expression induced resistance to treatment with Casodex, via decrease in apoptosis. Our studies further indicate that ACSL4 upregulates distinct pathway proteins including p-AKT, LSD1 and β-catenin. These results suggest ACSL4 could serve as a biomarker and potential therapeutic target for CRPC.

## INTRODUCTION

Although, androgen ablation therapy remains the standard of treatment for recurrent and metastatic PCa, most cases treated with ablation therapy will evolve into castration-resistant prostate cancer (CRPC), the primary cause of prostate cancer-related death. Importantly, the failure of androgen deprivation therapy is not accompanied by the loss of androgen receptor (AR) expression or transcriptional activity, and AR activity remains critical for tumor growth in CRPC [1]. AR expression is typically increased in CRPC [1], with restoration of AR activity through a variety of mechanisms including AR amplification and overexpression, AR mutation (mostly in the ligand-binding domain, conferring ligand promiscuity), increased intratumoral androgen synthesis, androgen-independent AR activation by cytokines and growth factors and constitutively active AR splice variants.

Increased expression of the fatty acid biosynthetic enzymes ATP: citrate lyase (ACLY), acetyl Co-A carboxylase (ACC) and fatty acid synthase (FASN) in a variety of tumors, including those that develop in prostate tissue, suggests a role for altered lipid metabolism in the genesis of a malignant phenotype [2]. *De novo* synthesis of free fatty acids and subsequent metabolic events, such as glycerolipid synthesis and β-oxidation, requires activation through condensation with a molecule of Coenzyme A (CoA). The enzymes responsible for the activation reaction comprise a family of proteins known as fatty acyl-CoA synthetases that are classified according to the chain length of their preferred substrates (short, medium, long, and very long) [3]. ACSL4 is a long-chain fatty acyl-CoA synthetase with a marked preference for arachidonic and eicosapentaenoic acid as substrates [4, 5]. Interestingly, ACSL4 is overexpressed in colon and liver cancer specimens compared to its low level expression in benign colon and liver [6-8]. Previous work from our laboratory has demonstrated an inverse relationship between the expression of ACSL4 and AR/ER in breast cancer cell lines and tissue samples; the data further suggested that co-expression of both a receptor and ACSL4 was indicative of hormone-independent growth [9, 10]. In ER-negative breast tumor samples, high ACSL4 expression predicted a shorter time to distant metastases [9] and was highest in triple negative breast cancer cell lines and tumor samples that lacked AR receptors [10]. With respect to function, we and others have demonstrated that forced expression of ACSL4 in ER-positive MCF7 cells results in increased proliferation, migration and invasion *in vitro* as well as increased growth in *in vivo* xenograft models [10-12]. These data raise the question of the function of ACSL4 enzyme activity in mediating the aggressive phenotype associated with hormone independence in PCa.

In this study, we investigate the function of ACSL4 in human PCa cell proliferation and invasion. Our results indicate that ACSL4 expression is able to induce a more aggressive phenotype of PCa and may be useful as a biomarker for castration resistance and/or a target for treatment.

## RESULTS

### Expression of ACSL4 in PCa cells

**Figure 1:**
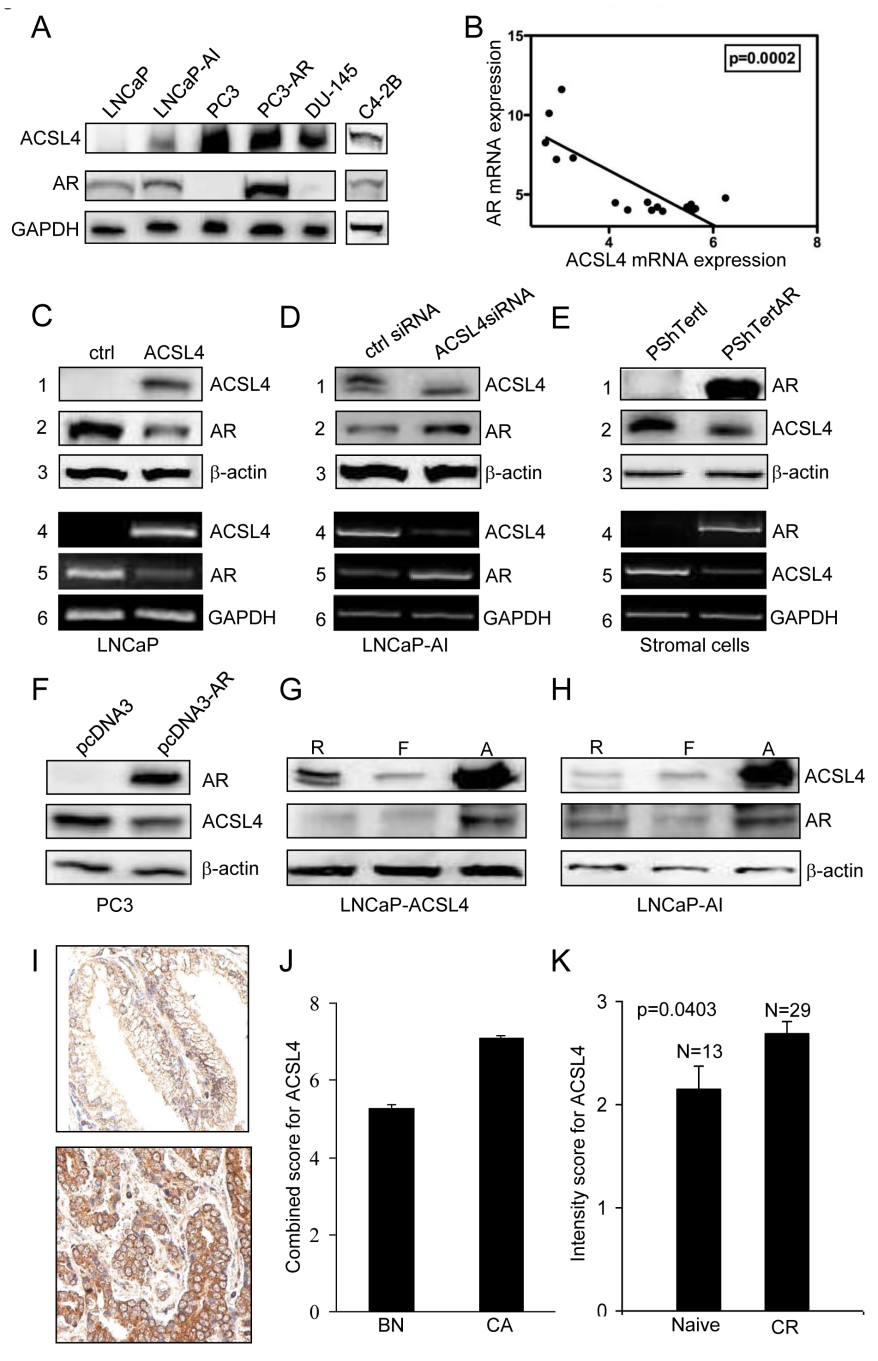
Expression of ACSL4 in PCa cell lines Western blot analysis of whole cell lysates showed expression of ACSL4 and AR with GAPDH as loading control **A.** Values shown are derived from mRNA expression data reported by Wang et al. [43]. **B.** ACSL4 and AR level in LNCaP ACSL4 overexpression cells and vector control cells by western blot analysis (1-3) with β-actin as loading control and RT-PCR analysis (4-6) with GAPDH as loading control **C.** ACSL4 and AR level in LNCaP-AI cells treated with ACSL4 siRNA and control siRNA by western blot analysis (1-3) with β-actin as loading control and RT-PCR analysis (4-6) with GAPDH as loading control **D.** ACSL4 and AR level in PShTertAR prostate stromal cells and PShTert prostate stromal cells by western blot analysis (1-3) with β-actin as loading control and RT-PCR analysis (4-6) with GAPDH as loading control **E.** ACSL4 and AR level in PC3 AR overexpression cells and PC3 cells by western blot analysis with β-actin as loading control **F.** ACSL4 and AR level in LNCaP ACSL4 overexpression cells in regular, hormone-free and androgen media by western blot analysis with β-actin as loading control **G.** ACSL4 and AR level in LNCaP-AI cells in regular, hormone-free and androgen media by western blot analysis with β-actin as loading control **H.** The expression of cytoplasmic ACSL4 in benign and malignant prostatic tissues by immunohistochemical staining **I.** The mean expression Allred score of cytoplasmic ACSL4 in benign and malignant prostatic tissues **J.** The intensity scores of expression of ACSL4 in hormone-naive PCa and hormone-resistant PCa **K.**

As previously reported in both PCa cell lines and tissue samples [9] there is an inverse relationship between ACSL4 and AR expression. Figure 1A extends this observation to additional cell lines. AR-positive, androgen-dependent LNCaP cells fail to express ACSL4, while AR-negative, androgen-independent PC3 and DU-145 cells express relatively high levels of ACSL4. AR-positive, androgen-independent LNCaP-AI and C4-2B cells express moderate levels of ACSL4. Figure 1B further illustrates the inverse relationship between AR and ACSL4 mRNA expression in a series of 16 PCa cell lines, as detailed in Table 1.

**Table 1:** The relative mRNA expression of AR and ACSL4 in a series of PCa and prostate epithelial cell lines

PCa Cell Lines	ACSL4	AR
PC3	5.60	4.00
DU145	4.93	4.22
LNCaP	2.78	8.26
22Rv	2.99	7.21
WPMV1	6.24	4.79
VCaP	2.85	10.12
MDAPCa2b	3.31	7.31
HPV7	4.74	4.51
HPV10	4.36	4.03
RWPE1	5.03	3.95
RWPE2	4.12	4.49
NB11	5.58	4.39
W99	5.66	4.11
PWR1E	4.82	4.02
DUCaP	3.09	11.62
NB26	5.50	4.22

To explore the dynamics of the inverse relationship between ACSL4 expression and AR, we first forced expression of ACSL4 in ACSL4-negative LNCaP cells. As demonstrated in Figure 1C, ectopic ACSL4 expression resulted in a decrease in AR expression at both the mRNA and protein levels. Alternatively, when ACSL4 expression in LNCaP-AI cells was abolished by treatment with siRNA, the expression of AR was increased (Figure 1D). This inverse relationship was also observed in stromal cells, which normally express ACSL4. Ecotopic AR expression in prostate stromal cells resulted in decreased ACSL4 expression at both the mRNA and protein levels (Figure 1E). Ectopic expression of AR in AR-negative PC3 cells likewise led to a reduction in ACSL4 expression (Figure 1F). Interestingly, ACSL4 and AR expression levels were increased when LNCaP-ACSL4 cells and LNCaP-AI cells were cultured in androgen-containing medium (Figure 1G and 1H). Similar observation is also found in VCaP cells (Supplemental Figure 1).

### Expression of ACSL4 in PCa tissue

We next studied the expression of ACSL4 protein in human PCa tissue samples by immunohistochemical analysis of a human PCa TMA derived from a cohort of PCa patients (*n* = 155) in various clinicopathological groups and benign prostate tissue (*n* = 124). We first examined the expression of cytoplasmic ACSL4 in benign and malignant prostatic epithelial cells (Figure 1I). The mean expression Allred score of cytoplasmic ACSL4 was increased in PCa by 1.3-fold compared to the benign tissues (*p* < 0.0001) (Figure 1J). A similar pattern was observed in the expression intensity score and percentage score (data not shown). Among the PCa patients with high stage (Stage III) PCa, ACSL4 expression percentage scores were significantly higher than those with a low stage (Stage I or II) cancer (*p* = 0.04). PCa patients with positive surgical margins showed increased ACSL4 expression intensity scores when compared to margin-negative cases (*p* = 0.04). The cases of hormone-resistant PCa showed significantly higher intensity scores for expression of ACSL4 in the cytoplasm compared to hormone-naive cases (*p* = 0.04) (Figure 1K), although the Allred score showed no significant difference. There was no significant difference in ACSL4 expression with respect to other clinicopathologic parameters studied, including age, Gleason score, PSA level and recurrence, and clinical follow-up. Finally, ACSL4 expression levels in African American patients are significantly higher than those in Caucasian patients (*p* = 0.04) (Supplemental Figure 2).

### ACSL4 promotes PCa growth and invasion

**Figure 2:**
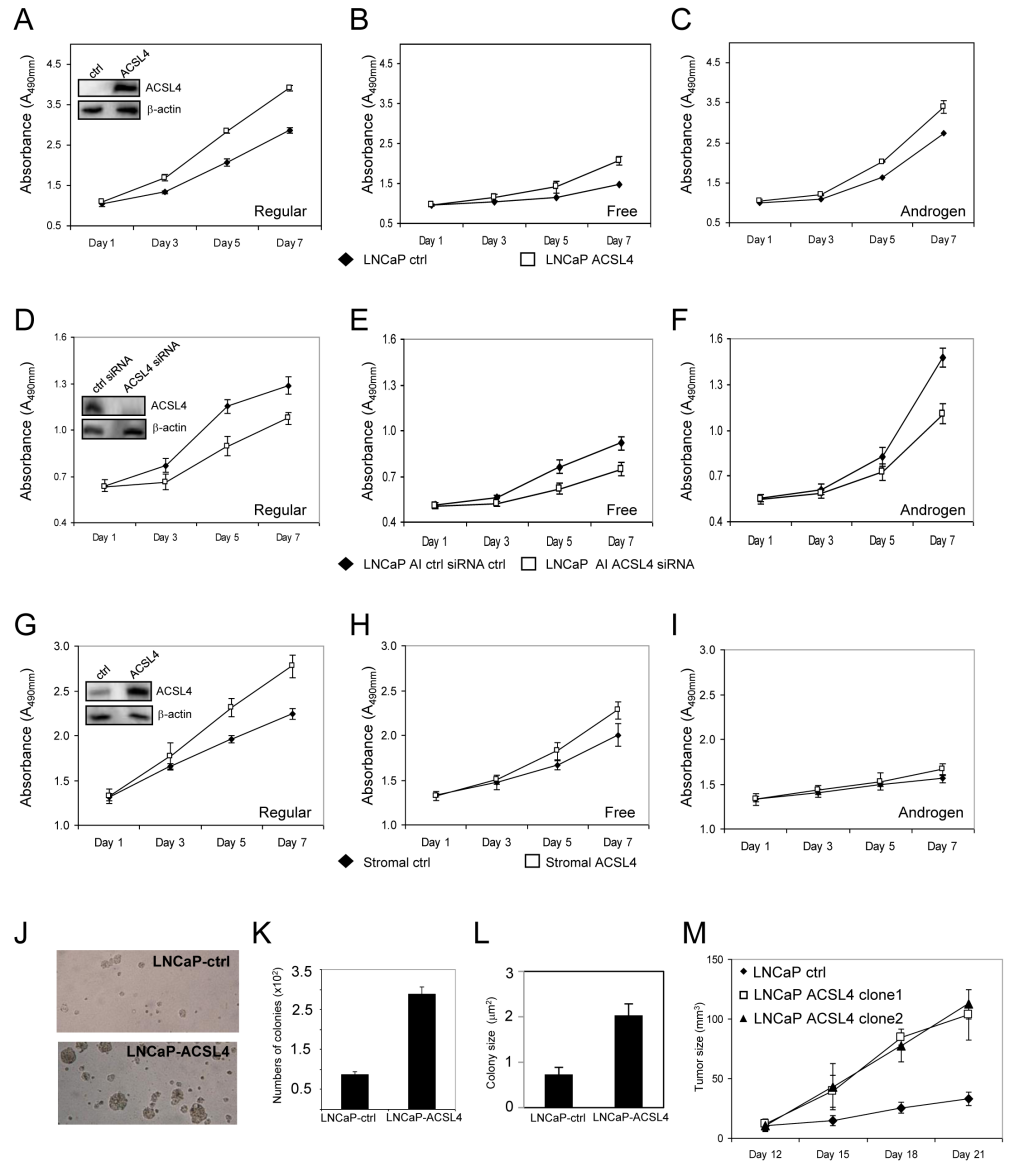
Effects of ACSL4 expression on PCa proliferation in regular, free and androgen medium Comparison of proliferation of vector control cells with ACSL4-overexpression LNCaP cells grown in regular, hormone-free and androgen media **A.**-**C.** The insert indicates ACSL4 expression in LNCaP ACSL4 overexpression cells and vector control cells with western blot and β-actin as loading control. Comparison of proliferation of LNCaP-AI cells treated with control siRNA and ACSL4 siRNA grown in regular, hormone-free and androgen **D.**-**F.** The insert indicates ACSL4 expression in LNCaP-AI cells treated with control siRNA and ACSL4 siRNA. Comparison of proliferation of prostate stromal cells with ACSL4 overexpression and vector control grown in regular, hormone-free, and androgen media **G.**-**I.** The insert indicates ACSL4 expression in prostate stromal with western blot and β-actin as loading control. Effects of ACSL4 overexpression on LNCaP by anchorage-independent assays **J.**-**L.** The *in vivo* growth of ACSL4-LNCaP cells tumors in nude mice xenografts. The graph indicates the growth of the tumor of one control cell line and two independent clonal cell lines with stable expression of ACSL4 **M.**

As shown in Figure 1A, LNCaP cells do not endogenously express ACSL4 protein. To examine the effect of ACSL4 expression in LNCaP cells, we utilized a Lentiviral expression system to create LNCaP-ACSL4 cells. When compared with control cells, LNCaP-ACSL4 cells exhibited an increased rate of proliferation in complete medium (*p* < 0.0001), as well as in hormone-free medium without (*p* < 0.0001), or with (*p* < 0.0001), added androgen (Figure 2A-2C). Additionally, we measured the effect of decreasing ACSL4 expression by siRNA treatment in the ACSL4-positive, AR positive androgen-independent, LNCaP-AI cells. When endogenous ACSL4 expression is ablated, the rate of proliferation is decreased compared with siRNA controls under all conditions (*p* < 0.0001 in complete medium, androgen-supplemented medium and hormone-free medium) (Figure 2D-2F).

Prostate stromal cells [13] normally express ACSL4 (Figure 1E), so we next questioned whether expression of ACSL4 in prostate stromal cells might influence the growth of neighboring tumor cells through paracrine signaling, possibly such as by prostaglandin E2 (PGE2) production [14]. We first established a stable prostate stromal cell line with overexpression of ACSL4 to study its effects on the proliferation of prostate stromal cells under the same conditions described for previous experiments with LNCaP and LNCaP-AI cells. Results were similar to those observed in LNCaP cells, but effects were reduced in magnitude, which is not surprising since stromal cells express endogenous ACSL4 (Figure 2G-2I). Pro-proliferative effects of ACSL4 in stromal cells were greatest in complete medium (*p* < 0.0001). To determine if ACSL4 expression in prostate stromal cells affects neighboring PCa cell growth *in vitro*, we performed proliferation assays using transwell indirect co-culture assays of stromal ACSL4 and control cells with PC3 and LNCaP cells. No significant effect on PCa growth was observed (*p* = 0.339 and *p* = 0.619) (Supplemental Figure 3).

We next examined the effect of ACSL4 expression on the anchorage-independent growth of LNCaP cells. We observed that ACSL4 expression increased the number and size of colonies compared with the control vector-transfected LNCaP cells. As shown in Figure 2J-2L, there was a 3-fold increase in colony number and colony size in ACSL4-expressing LNCaP cells (*p* < 0.0001).

To determine whether the positive growth regulatory effects of ACLS4 expression on LNCaP cells could be observed *in vivo*, we performed subcutaneous tumor xenograft experiments using immunodeficient nude mice. We observed a significantly increased rate of tumor growth in LNCaP xenografts expressing ACSL4 (*n* = 10 mice per group per clone) (Figure 2M) compared to vector controls (*n* = 10) (*p* < 0.0001) consistent with the data derived from *in vitro* experiments.

**Figure 3:**
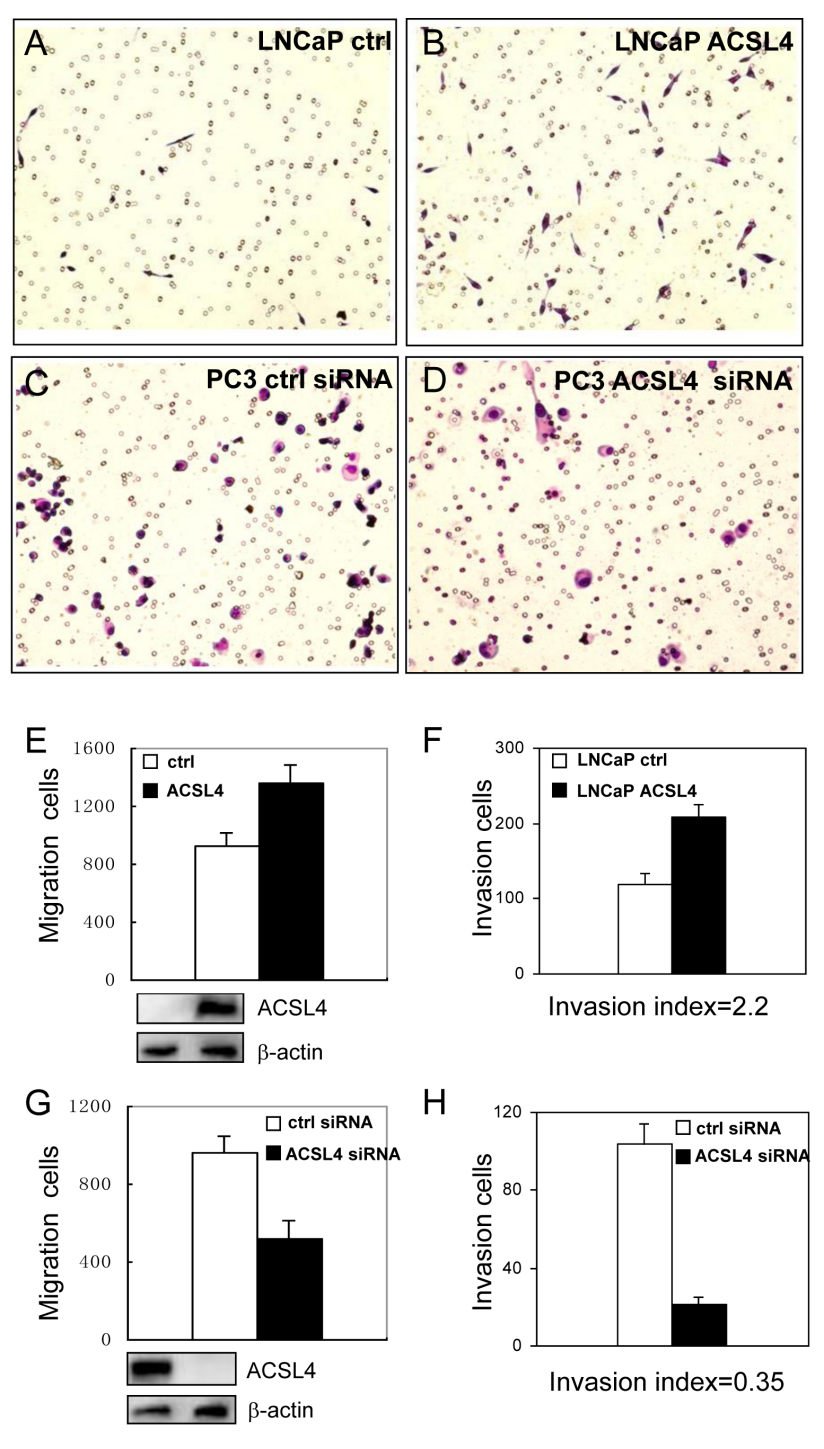
Effect of ACSL4 expression on invasion capability of PCa cells The migration and invasion ability were measured on LNCaP-ctrl and LNCaP-ACSL4 cells by Matrigel assay **A.**-**B.** Bar graph indicates the average number of cells per field, and Western blot analysis of ACSL4 expression in control and ACSL4-transfected LNCaP cells **E.**-**F.** PC3 cells were treated with ACSL4 siRNA or control siRNA **C.**-**D.** Bar graph indicates the average number of cells per field that traversed the membrane and Western blot analysis of ACSL4 expression in control and siRNA-treated cells **G.**-**H.**

We next examined the migration and invasion ability of LNCaP cells stably transfected with ACSL4. We observed increased migration of LNCaP-ACSL4 cells compared to control cells (Figure 3E). Increased ACSL4 expression also increased the number of invading cells by Matrigel invasion assay (Figure 3A, 3B and 3F). The invasion index, determined by percentage of invasion of test cells (LNCaP-ACSL4) over that of control cells (LNCaP), was 2.2. When ACSL4-positive PC3 cells were treated with ACSL4 siRNA, the migration and invasion capabilities were decreased significantly (Figure 3C, 3D, 3G and 3H). The invasion index was 0.35.

### High levels of ACSL4 mediate CRPC

**Figure 4:**
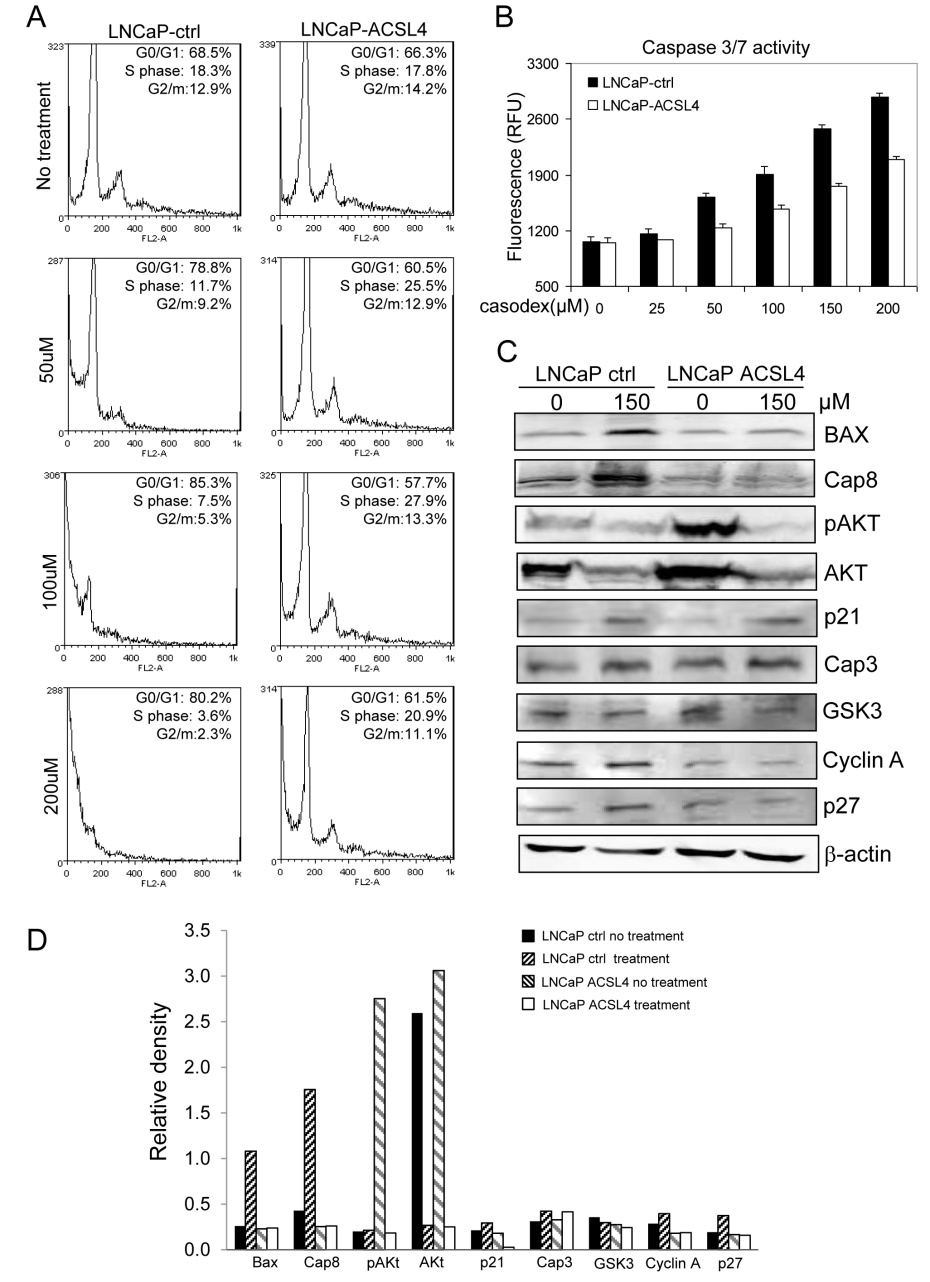
Effect of ACSL4 expression on apoptosis in LNCaP cells LNCaP cells were treated with different doses of Casodex and cell cycle was measured by propidium iodide staining and flow cytometry showing increased pre-G1/G0 population in dosage dependent manner **A.** Caspase 3/7 activity was detected on LNCaP-ACSL4 and LNCaP vector control cells with increasing concentrations of Casodex treatment **B.** The expression of apoptotic and cell cycle proteins in LNCaP-ACSL4 cells and control cells with and without Casodex treatment **C.** Bar graph indicates the relative quanitity of proteins on LNCaP-ACSL4 cells and LNCaP-ctrl cells with or without Casodex treatment by densitometric analysis **D.**

To elucidate the effect of ACSL4 on anti-androgen therapy, we first treated LNCaP control and ACSL4-expressing cells with Casodex at different concentrations followed by flow cytometry-based cell cycle analysis. As shown in Figure 4A, ACSL4 expression reduced the sensitivity of LNCaP cells to the effects of Casodex, inhibiting the ability of Casodex to block entry into the S phase of the cell cycle. Interestingly, there were fewer pre-G1 apoptotic cells in LNCaP cells expressing ACSL4 as a result of Casodex treatment. The ability of ACSL4 to reduce apoptosis induced by Casodex treatment was confirmed by measuring apoptosis using the Apo-ONE ^®^ Homogeneous Caspase-3/7 Assay (Figure 4B). The ability of Casodex to induce apoptosis has previously been shown to involve BAX and Caspase-8 [15] and the effect on the cell cycle appears to involve Cdc6, cyclin A, and cyclin B [16]. A decrease in Akt phosphorylation in PCa cells may mediate the mechanism of action of Casodex [17]. These cell cycle and apoptotic related proteins were analyzed via immunoblot after Casodex treatment at 150 μM on LNCaP-control and LNCaP-ACSL4 cells. As shown in Figure 4C, the ability of Casodex to increase BAX and CASP-8 expression was abolished as a result of ACSL4 expression. In addition, ACSL4 expression reduced CASP-8 expression even in the absence of Casodex. Interestingly, AKT phosphorylation was significantly increased as a result of ACSL4 expression, although there did not appear to be any difference in the ability of Casodex to inhibit either AKT expression or phosphorylation.

### Pathway proteins affected by ACSL4

**Figure 5:**
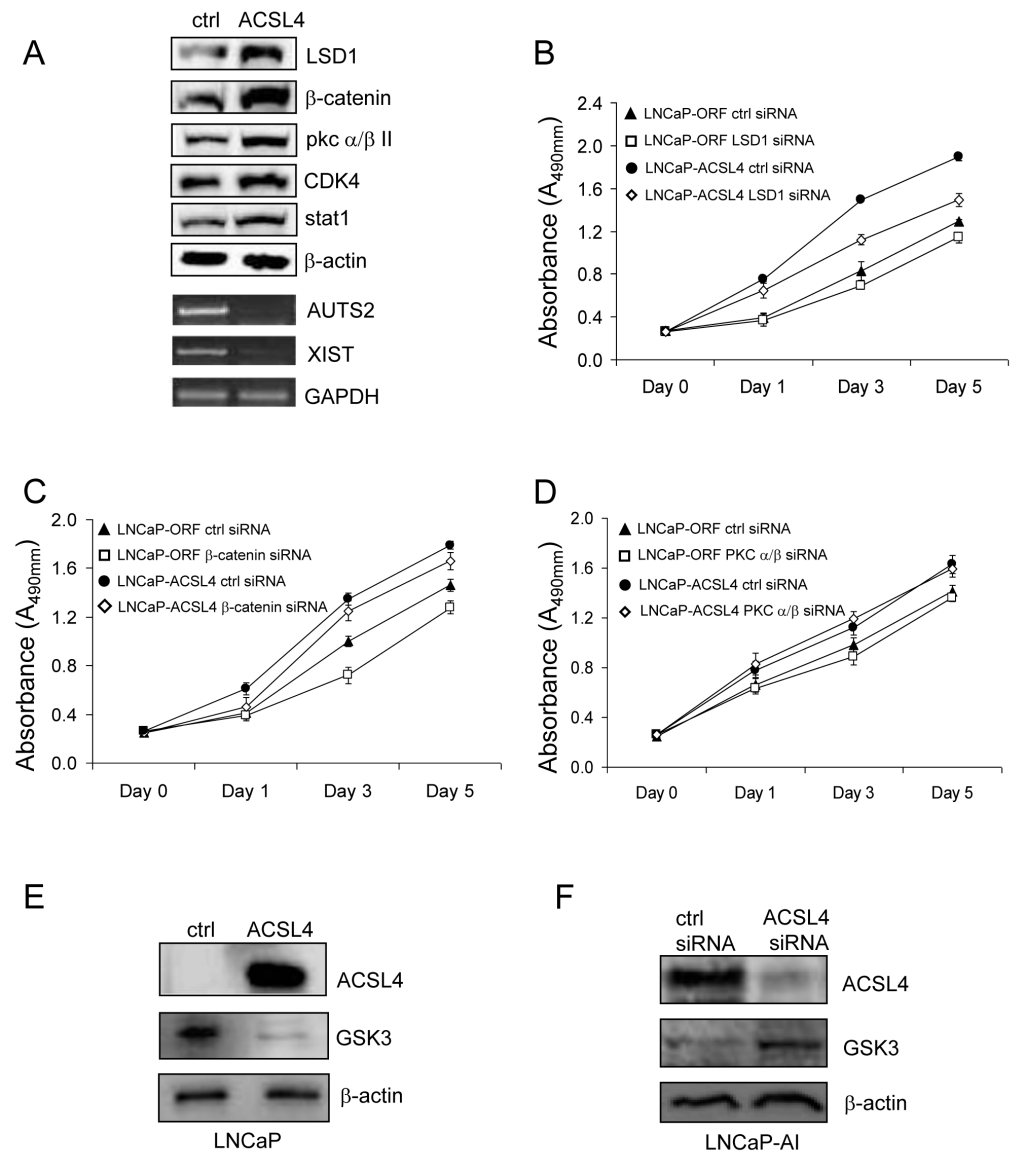
Western blot analysis of selected pathway proteins and their regulation of PCa cell proliferation Western blot was performed to confirm the expression level of proteins which increase in LNCaP-ACSL4 cells with PPAA **A.** The proliferation of LNCaP-ACSL4 cells after treatment with LSD1, β-catenin or PKC α/β siRNA compared to control siRNA **B.**-**D.** GSK3 expression level was decreased on LNCaP-ACSL4 cells compared with LNCaP-ctrl cells **E.** GSK3 expression level was increased in LNCaP-AI cells after treatment with ACSL4 siRNA **F.**

To explore the effect of ACSL4 on pathway proteins at a global level in PCa cells, we further evaluated protein expression as a function of ACSL4 expression using Protein Pathway Array Analysis (PPAA) to compare LNCaP control and LNCaP ACSL4-expressing cells [18]. Results are shown in Table 2. Of 199 pathway proteins examined [19], there were 9 proteins with increased and 10 proteins with decreased expression and/or phosphorylation levels. For example, there was a 5.26-fold increase in the level of LSD1 (Lysine-specific histone demethylase 1), a 4.98-fold increase in β-catenin, and more than two-fold increase in the levels of p-PKC α/βII, CDK4, HIF-3α, ADH, Calretinin and EGFR. We confirmed the changes in protein expression level for these findings by western blot (Figure 5A). Additionally, we previously reported decreased XIST and AUTS2 mRNA expression in breast cancer cells as a result of forced ACSL4 expression [10]. When we assessed the expression of XIST and AUTS2 in PCa in relation to ACSL4, we observed that both XIST and AUTS2 mRNA were decreased in LNCaP cells expressing ACSL4 (Figure 5A).

**Table 2:** Change in signal pathway proteins with increased ACSL4 in LNCaP cells (LNCaP-ACSL4/LNCaP-ctrl)

Protein	Fold Change
LSD1 (1B2E5)	**5.26**
β-catenin	**4.98**
HIF-3α	4.00
ADH	2.74
Calretinin	2.43
EGFR (1005)	**2.34**
p-PKC α/βII (Thr638/641)	**2.08**
Cdk4(C-22)	**2.07**
Stat1 (42H3)	1.66
Hsp90 (AC88)	−0.49
cyclin B1 (H-20)	−0.47
p38β (A-12)	−0.44
NFkBp50	−0.44
cdc25B (H-85)	−0.40
WT1 (C-19)	−0.38
PEDF	−0.38
CHK1 (G-4)	**−0.31**
Raf-B (F-3)	−0.28
alpha-tubulin (B-7)	−0.22

To determine the functional relevance of some of these protein alterations with respect to the proliferative action of ACSL4 on PCa, we examined the effect of ablating these proteins on the growth of PCa cells expressing high levels of ACSL4. The results shown in Figure 5B indicate that reduction in LSD1 caused a significant reduction in the growth of LNCaP-ACSL4 cells (*p* < 0.0001) but no significant effect on LNCaP control cells. The proliferation of LNCaP-ACSL4 cells was also decreased after β-catenin siRNA treatment (*p* = 0.012) (Figure 5C); However, the same difference was observed in control LNCaP cells, so this effect could be independent of ACSL4. There was no difference observed in proliferation in either LNCaP-ACSL4 cells or control cells as a result of siRNA-induced downregulation of PKC /β (Figure 5D). The GSK3 expression level was decreased in LNCaP-ACSL4 cells compared with the LNCaP vector control cells (Figure 5E), while the GSK3 expression level was increased in LNCaP-AI cells treated with ACSL4 specific siRNA (Figure 5F).

## DISCUSSION

The malignant transformation of normal cells, including prostatic epithelial cells, is accompanied by alterations in intermediary metabolism, including increased glucose utilization and enhanced lipogenesis. To date, attempts to exploit enhanced lipogenesis in the development of treatment modalities have yielded limited clinical application [20]. Previous studies have primarily focused on the fatty acid synthetic enzyme, FASN, as a potential target [21].

In this study we identify the fatty acid activating enzyme, ACSL4, as a potential biomarker for CRPC and target for treatment in a subset of PCa. The long-chain fatty-acyl-CoA synthetase family of 5 isoenzymes functions to activate long-chain fatty acids prior to their utilization, either as substrates for triglyceride and phospholipid synthesis, or as substrates for β oxidation. Previous studies, both *in vitro* [7-12, 22-24] and *in vivo* [25], have demonstrated that inhibition of the activity of these enzymes inhibits growth and survival of cancer cells. We have previously demonstrated an inverse relationship between sex steroid receptor(s) (AR and ER) expression and ACSL4 expression in both breast cancer and PCa [9]. The data with respect to breast cancer suggest that ACSL4, when co-expressed with estrogen receptor, is indicative of resistance to hormone-based targeted treatments in breast cancer [10]. ACSL4 expression in PCa cell lines is inversely associated with expression of AR, and, when co-expressed with AR correlates with androgen independent growth (Figure 1A and 1B). Furthermore, forced expression of ACSL4 in AR-positive, ACSL4-negative LNCaP cells results in a reduced expression of AR (Figure 1C), while inhibition of ACSL4 expression in LNCaP-AI cells results in increased AR expression. Conversely, forced expression of AR in ACSL4-positive, AR-negative PC3 cells causes a decrease in ACSL4 expression (Figure 1F). Interestingly, in cells co-expressing AR and ACSL4 (LNCaP-ACSL4 and LNCaP-AI) treatment with androgens increased both ACSL4 and AR expression (Figure 1G and 1H). The overall pattern of expression of ACSL4 in PCa cell lines indicates that ACSL4 expression is associated with a more aggressive phenotype. Of note, androgen treatment induces the expression of both ACSL4 and AR in LNCaP-AI and VCaP cells. The simultaneous increase in AR and ACSL4 may represent underlying drive of androgen ablation resistance though the mechanism remains to be determined. Assessment of ACSL4 expression in human PCa tissue samples supports this notion (Figure 1I-1K) that expression is increased in castration resistant as compared with hormone naive samples.

We assessed the effects of ACSL4 expression on cell functions by either forcing expression in ACSL4-negative LNCaP cells, or inhibiting expression in ACSL4-positive LNCaP-AI cells. Under all growth conditions (complete media or charcoaled-stripped media with or without androgen) expression of ACSL4 conferred a growth advantage (Figure 2). This proliferative advantage was also observed for anchorage-independent growth with increased colony size and number (Figure 2). Similar results were observed *in vivo* (Figure 2M). Cell migration and invasion were likewise enhanced by expression of ACSL4 (Figure 3). The growth advantage that resulted from ACSL4 expression is also accompanied by an increased resistance to treatment with the anti-androgen, Casodex. LNCaP cells expressing ACSL4 failed to increase BAX and Caspase-8 expression in the presence of Casodex (Figure 4).

A proteomic pathway array analysis comparing LNCaP and LNCaP-ACSL4 cells indicated a number of signaling proteins were affected by ACSL4 expression (Table 2). We examined the effect of ablating three of these proteins (LSD1, β-catenin and PKC α/βII) on cell growth. We found LSD1 ablation to specifically reverse the growth promotion by ACSL4 in LNCaP cells (Figure 5B). Previous studies have demonstrated that LSD1 stimulates proliferation of both androgen-dependent and androgen-independent cell lines [26, 27], and combined with our results, indicate that ACSL4 may promote proliferation by activation of LSD1 in PCa cells.

ACSL4 has a preference for arachidonic acid as substrate and treatment of PC3 cells with arachidonic acid has previously been shown to result in increased AKT phosphorylation [28]. Together with our data showing increased ACSL4 leading to increased p-AKT, this suggests ACSL4 effects may be mediated through its enzymatic interaction with arachidonic acid. In both breast cancer cells [11] and human arterial smooth muscle cells [29] forced expression of ACSL4 results in increased accumulation of prostaglandin E2 (PGE2), and prostaglandins have been implicated in PCa [30, 31]. Tjandrawinata et al have reported that PGE2 increases growth of prostate cancer cells and upregulates expression of its own synthesizing enzyme, COX-2 [32]. Thus, the PGE2 that results from expression of ACSL4 activity might exert an autocrine effect on prostate cancer cells, interacting with surface receptors to activate PI3K/pAKT [33]. The increased level of pAKT might then function to down-regulate AR expression, as has been shown previously [34-36].

In summary, we have presented data that confirm an association between AR expression and ACSL4 expression in human PCa. More specifically, we have provided evidence that ACSL4 expression is more closely associated with hormone-independent growth and may be a useful marker in determining response to hormonal therapy. In addition, it may be possible to target ACSL4 in the treatment of CRPC.

## MATERIALS AND METHODS

### Cell culture, flow cytometry, cell proliferation, anchorage-independent cell growth and *in vitro* Matrigel invasion assays

LNCaP cells were maintained in RPMI 1640 (Life Technologies) with 10% heat-inactivated bovine serum (fetal bovine serum). LNCaP-ACSL4 stable cells were constructed by lentiviral infection, using lentiviral-ACSL4 viral particles purchased from Invitrogen. The virus-containing solution was added to LNCaP cells and the stable cells were selected. Immunoblot was performed to demonstrate ACSL4 overexpression.

Cell cycle analysis was performed on FACSCalibur flow cytometer (BD Biosciences) and analyzed using Weasel software (The Walter and Eliza Hall Institute of Medical Research, Melbourne, Australia). Cells were prepared for flow cytometry as described previously [37]. Invasion assays were performed using BD Matrigel invasion chambers and 5% FBS was used as the chemoattractant in the lower chamber. Cells (5 × 10^4^) were incubated for 24 h before they were stained and counted. For invasion assays, the percentage of invasion was expressed as the ratio of invading cells over cell number normalized on day 2 of the growth curve.

Cell proliferation and invasion kinetics were measured by WST assay (Roche), and Matrigel invasion assays were performed as described previously [37-39]. For cell proliferation assays, regular medium is RPMI1640 containing phenol red, plus 10% fetal bovine serum, hormone free medium is phenol red-free RPMI1640 plus 10% charcoal stripped fetal bovine serum, and androgen medium is phenol red-free RPMI1640 plus 10% charcoal stripped fetal bovine serum containing 10 nM R1881. For co-culture experiments [13], epithelial cells were grown in Transwell inserts in 24-well plates above the stromal cells seeded on the lower plate. All experiments were performed in triplicate. The number of cells was counted every other day for 8 days.

Anchorage-independent cell growth in soft agar was performed in triplicate with cells (4×10^4^) suspended in 2ml of medium containing 0.35% agar (Becton Dickinson) spread on top of 5ml of 0.7% solidified agar. Total numbers of colonies were counted.

### Small interfering RNA knockdown of ACSL4

Cells were plated in T-25 flasks in complete medium lacking antibiotic and allowed to attach overnight. Cell densities at the start of the experiment were between 30% and 60% confluency. Transfection of small interfering RNA (siRNA; either control or ACSL4-specific Smart Pool siRNA purchased from Dharmacon, Lafayette, CO) into cells was accomplished using Lipofectamine RNAiMAX (Invitrogen) according to the protocol recommended by the manufacturer. Transfections were tested after 48 hour incubation.

### Immunoblot analysis

Whole cell extracts were subjected to SDS-PAGE and transferred to a nitrocellulose membrane for Western blot analysis. Blots were incubated with primary antibodies (AR, ER-α, and β-actin, Cell Signaling Technology, Inc.; ACSL4, Epitomic Inc) overnight at 4° C, washed with TBS-T, and incubated for 1 hour with the horseradish peroxidase-conjugated secondary antibody (1:5000; Amersham Biosciences). The protein bands were detected by an enhanced chemiluminescence kit (Amersham Biosciences). GAPDH and β-actin served as reference genes for normalization of ACSL4 expression.

### Caspase activity assay

To determine the effect of ACSL4 on apoptosis after LNCaP cells were treated with Casodex, the cells were subjected to Caspase 3/7 activity measurement with Caspase-Glo assay kit (Promega, Madison USA). Briefly, the plates containing cells were removed from the incubator and allowed to equilibrate to room temperature for 30 minutes. 100 μl of Caspase-Glo reagent was added to each well, the content of the well was gently mixed with a plate shaker at 300-500 rpm for 30 seconds. The plate was then incubated at room temperature for 8 hours. The luminescence of each sample was measured in a plate-reading luminometer (Thermo Labsystems) with parameters of 1 minute lag time and 0.5 second/well read time. The experiments were performed in triplicate and repeated on two separately-initiated cultures.

### Protein pathway array analysis (PPAA)

PPA analysis was performed as described previously [40], and antibodies were obtained from a number of sources [19]. The blot was hybridized with secondary horseradish peroxidase-conjugated antibodies (Bio-Rad) and chemiluminescence signal was detected using the ChemiDocXRS System. Differences in protein levels were analyzed by densitometric scanning and normalized using internal standards.

### Nude mice xenografts

Male nude mice (4 weeks old) were purchased from Charles River and maintained in accordance with the Institutional Animal Care and Use Committee-approved protocol. 1 × 10^7^cells were used for subcutaneous injection with Matrigel ECM (reconstituted basement membrane) to support tumor growth [37]. Each experimental group contained 10 mice. Tumors were measured with calipers every two days.

### PCa tissue microarray (TMA)

TMA of formalin-fixed, paraffin-embedded PCa were obtained from a cohort of 155 PCa patients managed by the New York University Langone Medical Center and the Department of Veterans Affairs New York Harbor Healthcare System (Manhattan), New York, NY between 1990 - 2005. The clinicopathological parameters were collected, including age, race, tumor stage and grade (Gleason score), PSA level and recurrence, margin status, and clinical follow-up. The hematoxylin-eosin stained slides were reviewed by two pathologists. Tumor staging was done according to the current American Joint Committee on Cancer (AJCC) staging criteria. The Gleason score and tumor staging were assigned according to modified criteria [41]. The benign controls (*n* = 124) were obtained from the benign prostatic tissue of PCa cases from the same cohort. The study was approved by NYU IRB under exempt category.

### Immunohistochemistry

Immunohistochemistry was performed using affinity-purified antibodies against ASCL4 (Santa Cruz, Dallas, TX). Paraffin-embedded tissue sections were dewaxed in xylene, rehydrated, and washed in Phosphate -buffered saline, pH 7.4. For antigen retrieval, paraffin sections were heated in a microwave oven (900 watts) in 10 mM citrate buffer followed by treatment with 3% H_2_O_2_ and blocking with 20% normal goat serum. Sections were then incubated with antibody against ASCL4 (1:100 dilution), followed by incubation with a biotinylated rabbit secondary antibody (1:1000, Vector Labs, Burlingame, CA) An avidin-biotin complex was formed and developed using diaminobenzidine chromagen, followed by a counter-stain with hematoxylin.

Immunohistochemical staining was examined and scored independently and in a blinded manner by two pathologists using the Allred immunohistochemistry score system [42]. Intensity levels, from 0 (as negative) to 3 (as strong), of cytoplasmic expression and percentage score, 0 (as 0%) to 5 (as 100%) were recorded semiquantitatively, resulting in a combined score for statistical analysis.

### Statistical analysis

The statistical analyses of the data were performed by using the two-way ANOVA. Differences were considered statistically significant if *p* < 0.05.

## SUPPLEMENTARY MATERIAL FIGURES


