# Supplementary material for: ACSL4 promotes prostate cancer growth, invasion and hormonal resistance
Source: Oncotarget. 2015 Nov 30;6(42):44849–63. doi: 10.18632/oncotarget.6438 (PMC4792596; doi:10.18632/oncotarget.6438)
Supplement: Supplementary file 1 [file oncotarget-06-44849-s001.pdf]

ACSL4 promotes prostate cancer growth, invasion and hormonal resistance

Supplementary Material

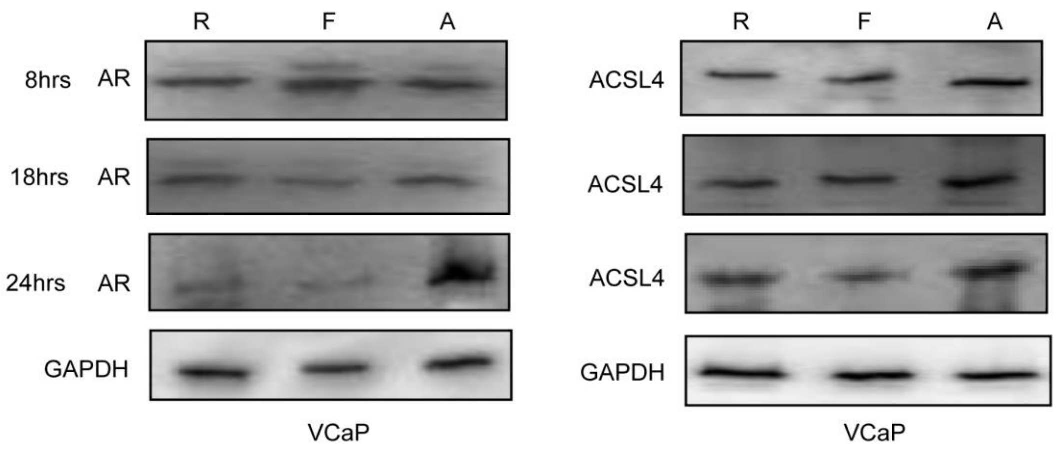

Supplementary Figure 1. AR and ACSL4 expression level in VCaP cells in different culture medium and at different culture times. AR and ACSL4 expression level in VCaP cells in complete, androgen free and androgen medium at 8, 18 and 24 hours culture.

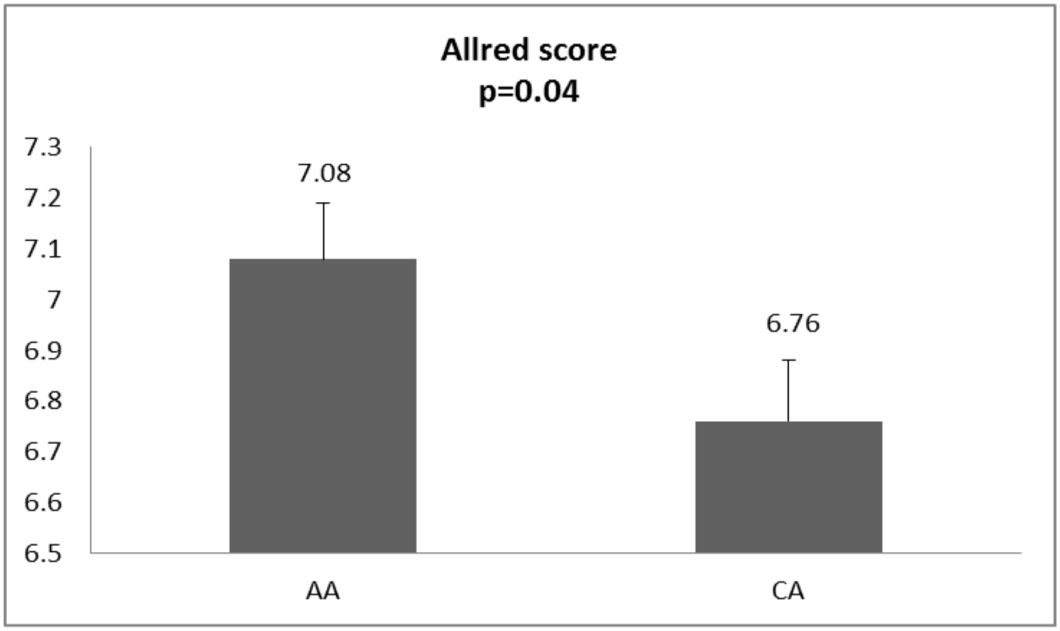

Supplementary Figure 2. Increased ACSL4 expression in African American (AA) prostate cancer compared to Caucasian American (CA) cases.

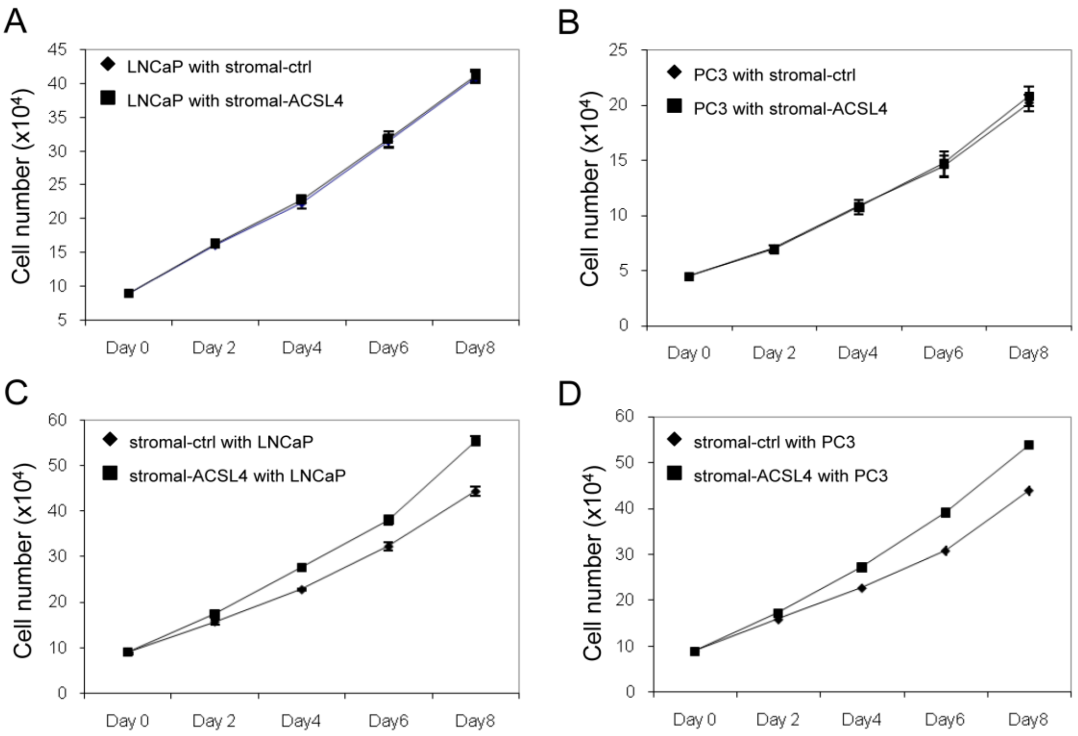

Supplementary Figure 3. The effects of prostate stromal-ACSL4 on PCa cell growth. Prostate stromal-ACSL4 stable cells were constructed by lentiviral infection. The proliferation of LNCaP cells when co-cultured with stromal-ACSL4 cells or stromal-ctrl cells (A); The proliferation of PC3 cells when co-cultured with stromal-ACSL4 cells or stromal-ctrl cells (B); The proliferation of stromal-ACSL4 cells and stromal-ctrl cells when co-cultured with LNCaP cells (C); The proliferation of stromal-ACSL cells and stromal-ctrl cells when co-cultured with PC3 cells (D).
